# Potential Role of SGLT‐2 Inhibitors in Improving Allograft Function and Reducing Rejection in Kidney Transplantation

**DOI:** 10.1111/ctr.70233

**Published:** 2025-09-02

**Authors:** Mehmet Emin Demir, Özant Helvacı, Tolga Yıldırım, Özgür Merhametsiz, Siren Sezer

**Affiliations:** ^1^ Department of Nephrology Atılım University Faculty of Medicine Ankara Turkey; ^2^ Department of Nephrology Gazi University Faculty of Medicine Ankara Turkey; ^3^ Department of Nephrology Hacettepe University Faculty of Medicine Ankara Turkey; ^4^ Department of Nephrology Yeni Yüzyıl University Faculty of Medicine Ankara Turkey

**Keywords:** immunosuppressant, mechanistic target of rapamycin (mTOR), rejection, signaling/signaling pathways

## Abstract

Sodium–glucose cotransporter‐2 inhibitors (SGLT‐2i) have demonstrated renoprotective and cardioprotective benefits beyond their antiglycemic effects. Their potential utility in kidney transplant recipients (KTRs) for preserving graft function and reducing rejection risk is currently under active investigation. Preliminary studies indicate that SGLT‐2i therapy stabilizes estimated glomerular filtration rate (eGFR), decreases glomerular hyperfiltration, and improves metabolic outcomes in KTRs. Emerging clinical evidence also suggests that SGLT‐2i may be associated with reduced rates of acute rejection, although direct immunosuppressive actions remain unclear. Experimental findings further suggest that SGLT‐2i modulates gene regulation pathways involved in inflammation, oxidative stress, and fibrosis, contributing to improved allograft outcomes. Current safety data in KTRs are reassuring, without significant increases in urinary tract infections or adverse graft events. Nevertheless, long‐term prospective studies specific to transplant populations are lacking. This review summarizes available evidence regarding the mechanisms of action, clinical efficacy, and safety profile of SGLT‐2i in kidney transplantation, emphasizing their metabolic, hemodynamic, inflammatory, and immunomodulatory effects.

## 1 Introduction

Organ transplantation is life‐saving for patients with end‐stage organ failure; however, long‐term allograft survival is often limited by immune‐mediated injury and metabolic complications of immunosuppressive therapy [1, 2]. Although early acute rejection episodes are associated with adverse outcomes regarding allograft function, late rejection episodes and adverse cardiometabolic events significantly impair long‐term transplant success [3, 4, 5, 6]. Ongoing research seeks novel therapies (e.g., belatacept, anti‐CD40 and anti‐CD154 antibodies, IL‐6/IL‐6R inhibitors, imlifidase) to extend graft longevity, including agents that might provide both immunomodulatory and metabolic benefits [7].

Sodium‐glucose cotransporter‐2 (SGLT‐2) inhibitors were initially developed as antihyperglycemic agents for type 2 diabetes mellitus (T2DM). However, growing evidence indicates their renal and cardiovascular benefits extend beyond glycemic control in diabetes and chronic kidney disease (CKD) (Table 1). Beyond glycemic control and enhanced tubuloglomerular feedback, SGLT‐2 inhibitors exhibit anti‐inflammatory, anti‐fibrotic, hemodynamic, and oxidative stress‐modulating properties, supporting their broader clinical use, including in transplantation [8, 9]. They also exert cardioprotective effects by acting directly on cardiomyocytes, independent of SGLT‐2 or renal tubule function, suggesting potential benefits for other organs and allografts [10, 11].

**TABLE 1:** Major clinical trials of SGLT‐2 inhibitors in chronic kidney disease.

Study (Year)	Population (CKD characteristics)	Number of participants	Intervention (vs. placebo)	Key renal outcomes
CREDENCE (2019)	T2DM with CKD (eGFR 30–90; ACR ≥ 300 mg/g)	4,401	Canagliflozin 100 mg daily	∼30% ↓ in progression to ESRD, doubling of Cr, or renal/CV death (HR 0.70, *p* < 0.001). Trial stopped early for efficacy.
DAPA‐CKD (2020)	CKD with or without T2DM (eGFR 25–75; ACR ≥ 200)	4,304	Dapagliflozin 10 mg daily	39%–44% ↓ in sustained ≥ 50% eGFR decline, ESRD, or renal/CV death (HR 0.56 for renal composite). Benefits also seen in nondiabetics.
EMPA‐Kidney (2022)	CKD with or without T2DM (eGFR 20–45, or up to 90 if ACR ≥ 200)	6,609	Empagliflozin 10 mg daily	28% ↓ in progression of CKD or CV death (HR 0.72, *p* < 0.0001). Included > 50% patients without diabetes; stopped early for efficacy.
SCORED (2020)	CKD with T2DM (eGFR 25–60, with or without albuminuria	10,584	Sotagliflozin 200 mg, uptitrated to 400 mg daily	Slowed eGFR decline and reduced albuminuria with SGLT‐2 inhibition

Abbreviations: ACR, albumin/creatinine ratio; CV, cardiovascular; Cr; creatinine; eGFR; estimated glomerular filtration rate; ESRD, end‐stage renal disease; T2DM; type  2 diabetes mellitus.

The use of SGLT‐2 inhibitors in KTRs, particularly for preserving allograft function or preventing rejection, is an evolving area of interest. Growing evidence supports their safety and efficacy in controlling hyperglycemia in diabetic KTRs [12, 13, 14]. While their direct impact on alloimmune responses is not yet clear, their ability to reduce glomerular hyperfiltration, improve blood pressure, and mitigate components of metabolic syndrome may indirectly enhance allograft outcomes. Given the high burden of cardiovascular and metabolic comorbidities in KTRs, SGLT‐2 inhibitors could provide multifaceted protection in transplant patients. However, their effects on immune function and rejection risk remain to be fully elucidated.

This review evaluates the potential of SGLT‐2 inhibitors to improve kidney allograft outcomes. We first summarize the mechanisms by which SGLT‐2 inhibitors exert renoprotective effects in native kidneys and the rationale for their use in KTRs. We then review the current clinical evidence in KTRs, including glycemic control, graft function, safety (with an emphasis on infection risk), and the potential link between SGLT‐2 inhibitors and rejection, and discuss how SGLT‐2 inhibitors might complement existing immunosuppressive regimens. Finally, we highlight gaps in knowledge and the need for future studies, especially randomized trials, to fully define the role of SGLT‐2 inhibitors in KTRs.

## 2 Materials and Methods

We conducted a comprehensive literature search using PubMed, Scopus, and Web of Science to identify studies on the use of SGLT‐2 inhibitors in kidney transplant recipients. The search covered publications up to April 15, 2025, with keywords including “SGLT‐2 inhibitors,” “kidney transplantation,” “allograft rejection,” “glomerular hyperfiltration,” “metabolic regulation,” and “immunomodulation.” We also manually reviewed reference lists of relevant articles to ensure inclusion of all pertinent studies.

Studies evaluating the effects of SGLT‐2 inhibitors on metabolic outcomes, allograft function, rejection, and safety (e.g., infections, acute kidney injury, diabetic ketoacidosis) in KTRs were included. Both clinical studies in humans and relevant animal studies were considered. Articles were excluded if they did not present original data (e.g., isolated case reports, editorials/commentaries without new data) or if they focused solely on nontransplant populations without extrapolation to KTRs. Given the heterogeneity of study designs and endpoints, we employed a narrative synthesis approach, emphasizing findings most relevant to allograft outcomes in KTRs. When interpreting small or observational studies, we noted their limitations (sample size, follow‐up, biases) to temper conclusions.

### 2.1 Role of Artificial Intelligence

We used AI‐based tools to assist with compiling literature and editing for clarity. Specifically, AI algorithms helped screen large volumes of text for relevant content and check language and grammar errors. The authors made all analyses, interpretations, and conclusions, and rigorously reviewed and revised the manuscript to ensure it accurately reflects the current evidence on SGLT‐2 inhibitors in transplantation.

## 3 Potential Benefits of Sodium–Glucose Cotransporter‐2 Inhibitors (SGLT‐2i) on The Native Kidney

### 3.1 Hemodynamic and Metabolic Effects in CKD

SGLT‐2i confer substantial renoprotective effects in CKD through multiple synergistic mechanisms. By promoting glycosuria and natriuresis, SGLT‐2i lowers plasma glucose and blood pressure, thereby reducing glomerular hyperfiltration. In diabetic kidney disease, they further restore tubuloglomerular feedback, decreasing intraglomerular pressure and mitigating hyperfiltration injury [15, 16, 17, 18]. Beyond these hemodynamic benefits, SGLT‐2 inhibitors exert beneficial cellular effects, reducing glucotoxicity and lipotoxicity by improving insulin sensitivity and lowering hyperglycemia. Additionally, their diuretic action might reduce interstitial congestion, enhancing peritubular capillary blood flow and kidney oxygenation [19]. Furthermore, SGLT‐2 inhibitors promote weight loss and facilitate a shift toward ketone metabolism, which may be more energy‐efficient for renal function [20]. Collectively, these mechanisms interrupt maladaptive cycles of nephron injury, stabilize GFR, and slow structural kidney damage.

Major cardiovascular outcome trials in type 2 diabetes, including EMPA‐REG OUTCOME, CANVAS, and DECLARE‐TIMI 58, have demonstrated significant renal benefits with SGLT‐2 inhibitors therapy, such as slower CKD progression and fewer cardiovascular events compared to placebo [15, 21, 22]. Dedicated CKD‐specific trials—DAPA‐CKD, CREDENCE, and EMPA‐KIDNEY, have further confirmed that SGLT‐2 inhibitors significantly reduce the risk of GFR decline, end‐stage renal disease, or death, even in patients without diabetes (Table 1) [16, 17, 23]. These findings have led to the inclusion of SGLT‐2 inhibitors in the 2022 KDIGO guidelines as part of standard CKD therapy [24].

### 3.2 Anti‐Inflammatory and Antioxidative Effects

SGLT‐2 inhibitors have been shown to attenuate renal inflammation and oxidative stress, which are common pathways in CKD progression. In preclinical studies with Zucker diabetic fatty (ZDF) rat models, empagliflozin reduced glucotoxicity and prevented endothelial dysfunction, lowered oxidative stress, and exhibited anti‐inflammatory effects despite persistent hyperlipidemia and hyperinsulinemia [25]. SGLT‐2 inhibitors may preserve vascular integrity in the kidney by restoring nitric oxide bioavailability and reducing endothelial dysfunction [26, 27]. Solini et al. found that 4 weeks of dapagliflozin therapy led to changes in circulating levels of certain microRNAs associated with inflammation, fibrosis, and vascular health [28]. Although chromatin‐level changes were not assessed in that study, the altered miRNA profile with dapagliflozin (e.g., reduced miR‐30e‐5p) suggests downstream anti‐inflammatory gene regulation in native kidneys, which may be relevant for vascular protection in transplant kidneys.

SGLT‐2i also interferes with pathways like NF‐κB and Nrf2 in the kidney. By reducing pro‐inflammatory signaling (NF‐κB) and enhancing antioxidant responses (Nrf2), they can decrease macrophage infiltration and fibrosis in renal tissue [29, 30]. These “pleiotropic” effects support the notion that SGLT‐2i therapy addresses not only hemodynamic stressors but also the injurious metabolic and immune environment in CKD.

### 3.3 Mammalian Target of Rapamycin (mTOR) Pathway and Metabolic Signaling

**FIGURE 1:**
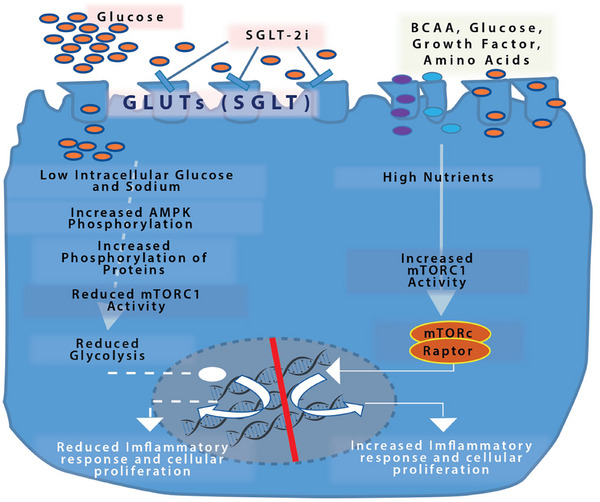
Nutrient‐sensing pathways modulated by SGLT‐2 inhibitors. SGLT‐2 inhibitors create a low‐nutrient state by promoting glucosuria and natriuresis, activating AMPK, and inhibiting mTORC1 signaling. This metabolic shift suppresses cell proliferation, glycolysis, and pro‐inflammatory responses, mimicking calorie restriction and providing protection against hypertrophy and inflammation. AMPK, AMP‐activated protein kinase; GLUT, glucose transporter; mTORC1, mammalian target of rapamycin complex 1; SGLT‐2i, sodium–glucose cotransporter‐2 inhibitors.

A key insight into SGLT‐2 inhibitors’ renoprotective mechanism is their interaction with nutrient‐sensing pathways, particularly the mTOR. mTOR exists in two complexes, mTORC1 and mTORC2, which regulate cell growth, autophagy, and metabolism in response to nutrients and growth factors. In diabetic kidney disease, hyperactivation of mTORC1 in podocytes and tubular cells contributes to glomerular hypertrophy, proteinuria, and fibrosis [31, 32]. SGLT‐2 inhibitors appear to dampen mTORC1 activity, likely by reducing intracellular glucose and amino acid availability (notably branched‐chain amino acids) in renal cells. Studies by Kogot‐Levin et al. demonstrated that SGLT‐2 inhibition leads to mTORC1 suppression in diabetic mice, and importantly, that pharmacologic or genetic mTORC1 inhibition can replicate the renoprotective effects of SGLT‐2 inhibitors, whereas forced mTORC1 activation diminishes those benefits (Figure 1) [33].

Additionally, SGLT‐2 inhibitors reduce renal cortical levels of toxic metabolic byproducts [34]. By promoting urinary excretion of glucose and diverting metabolism toward ketogenesis, SGLT‐2i decreases intracellular levels of glucose metabolites that feed the polyol and hexosamine pathways, key sources of oxidative stress in diabetes. They also reduce proximal tubular oxygen consumption (due to less sodium reabsorption and gluconeogenesis), which can relieve local hypoxia in the kidney cortex.

## 4 Impact of SGLT‐2 Inhibitors on Immune Cells and Alloimmunity

Recent research suggests that SGLT‐2i can alter the bioenergetics and function of T‐lymphocytes and other immune cells, potentially attenuating harmful alloimmune responses.

### 4.1 T‐Cell Metabolic Reprogramming

T cells require substantial metabolic reprogramming upon activation. Engagement of the T‐cell receptor and co‐stimulatory signals triggers a shift from oxidative phosphorylation to aerobic glycolysis (the Warburg effect), rapidly generating ATP and biosynthetic precursors necessary for clonal expansion [35, 36]. The PI3K‐Akt‐mTORC1 and c‐Myc pathways are pivotal for this metabolic transition, enhancing glucose uptake and glycolytic flux in activated T cells [37]. Consequently, targeting T‐cell metabolism emerges as a promising immunosuppressive strategy to modulate T‐cell activation and effector function [38, 39].

Jenkins et al. demonstrated that canagliflozin suppresses human T‐cell activation via metabolic interference, specifically by inhibiting both mTORC1 and c‐Myc, which reduces glucose uptake and glycolysis, inducing a T‐cell “energy crisis” [40]. Canagliflozin‐treated T cells exhibit decreased expression of activation markers and produce fewer pro‐inflammatory cytokines (IFN‐γ, IL‐2, IL‐17). Interestingly, these effects occur despite T cells not expressing SGLT‐2, suggesting canagliflozin acts through off‐target mechanisms, potentially via alternative glucose transporters, metabolic enzymes, or indirectly through systemic metabolites such as ketone bodies. Notably, similar immunometabolic effects have been observed with other SGLT‐2 inhibitors, suggesting a class‐wide phenomenon. For example, empagliflozin treatment in patients with type 2 diabetes decreased IL‐17‐producing T helper cells while promoting regulatory T‐cell differentiation, indicating a shift toward an anti‐inflammatory immune profile [41].

**FIGURE 2:**
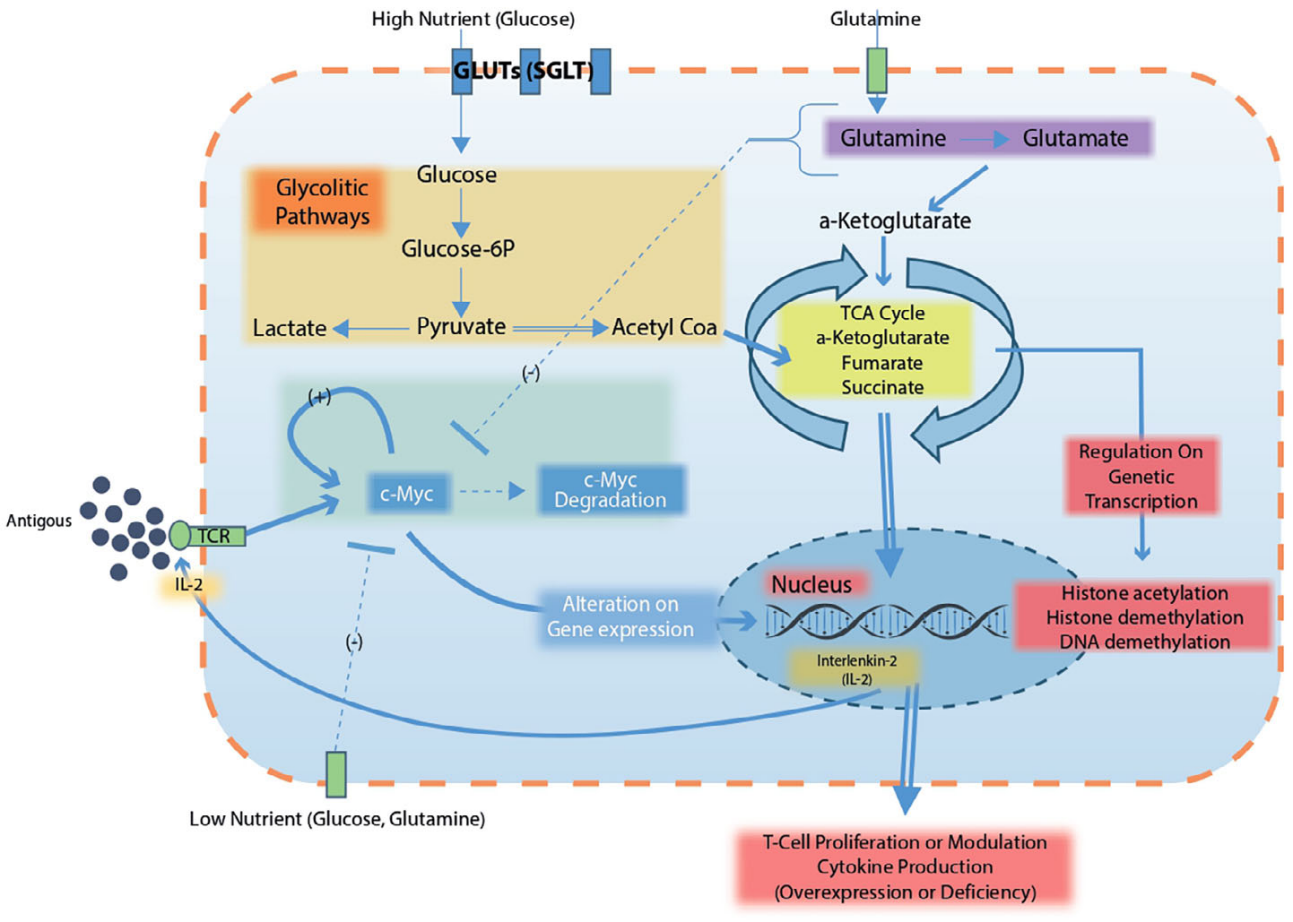
Metabolic modulation of T‐cell activation by SGLT‐2 inhibition. High‐nutrient conditions (left) support robust glycolysis and glutaminolysis, stabilizing c‐Myc, enhancing IL‐2 production, and driving T‐cell proliferation. Under low‐nutrient conditions induced by SGLT‐2 inhibitors (right), reduced glycolysis and c‐Myc degradation limit metabolite availability, attenuating histone acetylation and cytokine production. This metabolic shift suppresses T‐cell activation and proliferation, promoting a less inflammatory, more quiescent or regulatory phenotype. c‐Myc, MYC proto‐oncogene; GLUT, glucose transporter; IFN‐γ, interferon‐gamma; IL‐17, interleukin‐17; IL‐2, interleukin‐2; mTORC1, mechanistic target of rapamycin complex 1; SGLT‐2i, sodium–glucose cotransporter‐2 inhibitors.

Collectively, these findings suggest that beyond their well‐established metabolic and hemodynamic benefits, SGLT‐2 inhibitors may also modulate alloimmune responses. By selectively dampening pathogenic T‐cell activation and promoting regulatory immune responses, these agents could potentially reduce alloimmune reactivity and consequently lower the risk of graft rejection (Figure 2).

#### 4.1.1 Mechanistic Pathways Influenced

SGLT‐2i impacts several pathways in T cells:

- **Glycolysis and glutaminolysis**: Canagliflozin‐treated T cells show reduced glycolytic flux and glutamine utilization, leading to lower production of key metabolic intermediates (like acetyl‐CoA and α‐ketoglutarate) [40, 42, 43]. Empagliflozin modulates T‐cell metabolism by inhibiting glycolysis and enhancing oxidative phosphorylation in CD4⁺ T cells, thereby reducing Th1 and Th17 subsets and increasing regulatory T cells. These effects involve inhibition of the mTOR pathway, similar to mechanisms reported for canagliflozin [44].
- **c‐Myc Inhibition**: Recent studies showed that empagliflozin and canagliflozin inhibit c‐Myc‐driven hepatocellular carcinoma by targeting the mTOR pathway, reducing c‐Myc expression, glycolysis, and glutamine metabolism [40, 45].
- **Cytokine production**: Canagliflozin suppresses Th1/Th2/Th17 cytokine production (e.g., IFN‐γ, IL‐4, IL‐17) and decreases systemic inflammatory markers like IL‐6 and TNF‐α, suggesting broad anti‐inflammatory potential [40]. Similarly, dapagliflozin has been reported to reverse the imbalance between Th17 and regulatory T cells, suggesting its role in modulating immune responses [46].
- **T‐cell differentiation**: Canagliflozin alters CD4⁺ T‐cell lineage specification by downregulating transcription factors T‐bet (Th1), GATA3 (Th2), and RORγt (Th17), thereby reducing pro‐inflammatory Th1/Th2/Th17 populations [47]. This effect was associated with improved renal outcomes in models of immune‐mediated nephropathy [46, 47]. Empagliflozin similarly reduces IL‐17‐related pathways and promotes regulatory T cell differentiation, further supporting a class‐wide immunomodulatory role [41].
- **Cell survival**: By inducing an energy‐deprived state, canagliflozin can promote apoptosis of overly active T cells. It causes mitochondrial dysfunction in activated T cells, increasing mitochondrial ROS and depolarizing mitochondrial membranes, leading to selective apoptosis of high‐energy‐demand effector T cells. This effect might preferentially affect pathogenic effector T cells without harming resting or regulatory T cells [40].

Zhang et al. recently reviewed the broad anti‐inflammatory effects of SGLT‐2 inhibitors in patients with T2DM. They reported that various SGLT‐2 inhibitors (canagliflozin, empagliflozin, dapagliflozin, luseogliflozin, and ipragliflozin) all effectively reduce inflammatory biomarkers such as TNF‐α, IL‐6, IL‐1β, MCP‐1, and hsCRP [48]. Mechanistically, these agents modulate multiple pathways overlapping with what we described: inhibition of glycolysis, suppression of c‐Myc activity, reduction in cytokine production, modulation of T‐cell differentiation, and enhancement of apoptotic clearance of activated cells. They also suppress activation of the NLRP3 inflammasome, promote anti‐inflammatory (M2) macrophage polarization, activate AMPK, and improve metabolic parameters like hyperglycemia, hyperinsulinemia, uric acid levels, body weight, and ketone body production. While sotagliflozin (a dual SGLT1/2 inhibitor) potentially offers even broader metabolic and anti‐inflammatory effects, current experimental data for it are limited.

#### 4.1.2 Neutrophils and Other Immune Cells

There is evidence that SGLT‐2 inhibitors benefit extend to innate immune cells as well. Patients with glycogen storage disease type Ib (GSD‐Ib), who suffer from chronic neutropenia and neutrophil dysfunction due to a buildup of 1,5‐anhydroglucitol‐6‐phosphate, have been successfully treated with SGLT‐2 inhibitors. Empagliflozin causes urinary excretion of the offending metabolite, leading to restored neutrophil counts and improved neutrophil function [49]. While GSD‐Ib is a rare, specific scenario, it illustrates that SGLT‐2 inhibition can improve immune cell function (neutrophils, in this case) via metabolic modulation. In KTRs, robust neutrophil function is critical to prevent infections. The GSD‐Ib experience suggests that SGLT‐2i do not universally suppress immunity in a harmful way; instead, they may selectively dampen pathological immune activation (T cells) while preserving or even enhancing certain normal immune functions (like neutrophil‐mediated host defense under specific conditions).

## 5 Additional Mechanistic Insights

### 5.1 Gene Regulation and Oxygenation

Beyond the systemic and cellular effects discussed, SGLT‐2 inhibitors may confer additional organ‐protective benefits through influencing gene regulation and tissue oxygenation.

#### 5.1.1 Metabolic Regulation of Gene Expression

**FIGURE 3:**
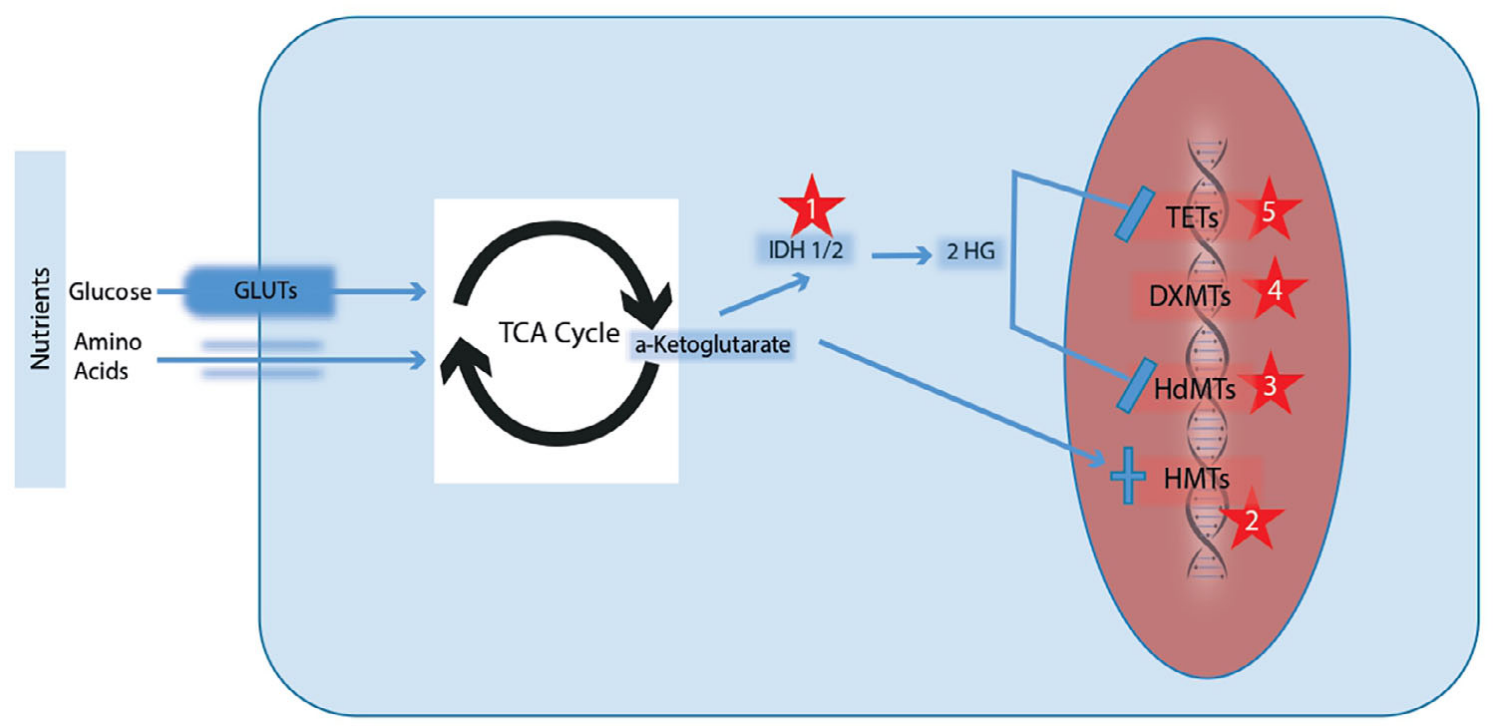
Nutrient‐driven epigenetic modulation in renal cells. High nutrient availability (glucose and amino acids) generates metabolites such as aKG, which can be converted into the oncometabolite 2HG via IDH1/2 enzymes. Elevated 2HG disrupts DNA/histone methylation and demethylation processes, causing aberrant gene expression. SGLT‐2 inhibitors reduce intracellular glucose, potentially limiting these epigenetic changes and maintaining normal gene expression in kidney cells. 2HG, 2‐hydroxyglutarate; aKG, α‐ketoglutarate; DNMT, DNA methyltransferase; GLUT, glucose transporter; HDMT, histone demethylase; HMT, histone methyltransferase; IDH1/2, isocitrate dehydrogenase 1/2; SGLT‐2i, sodium–glucose cotransporter‐2 inhibitors; TCA, tricarboxylic acid cycle; TET, ten–eleven translocation.

Cellular metabolism and gene expression are tightly linked. Changes in the availability of key metabolites can alter epigenetic marks (such as histone acetylation and DNA methylation) and transcription factor activity. SGLT‐2 inhibitors induced metabolic shifts may thus have downstream genomic effects:

- **Histone acetylation/methylation**: SGLT‐2 inhibitors reduce cytosolic glucose metabolism, decreasing acetyl‐CoA and S‐adenosylmethionine levels, substrates essential for histone acetylation and DNA/histone methylation (Figure 3). By limiting these metabolites, SGLT‐2 inhibitors may mitigate glucose‐associated aberrant epigenetic changes, potentially normalizing gene expression profiles. Similar effects have been suggested by Reid et al., who demonstrated nutrient deprivation can reverse diabetic kidney epigenetic alterations. Although direct evidence remains limited, this highlights a broader potential role for SGLT‐2 inhibitors in epigenetic and metabolic regulation [50].
- **Ketone‐related signaling**: SGLT‐2 inhibitors increase circulating ketone bodies such as β‐hydroxybutyrate (βOHB), which act not only as alternative fuels but also as epigenetic modulators. Elevated βOHB inhibits class I histone deacetylases (HDACs), enhancing histone acetylation and activating protective gene programs. Additionally, βOHB serves as a substrate for histone β‐hydroxybutyrylation, promoting expression of oxidative stress‐response genes. These epigenetic effects may mediate some renal benefits of SGLT‐2 inhibitors therapy by increasing resistance to oxidative stress and inflammation. Indeed, Nishitani et al. demonstrated increased βOHB and associated histone modifications in dapagliflozin‐treated diabetic mice, highlighting this epigenetic mechanism [51].

#### 5.1.2 Tissue Oxygenation

**FIGURE 4:**
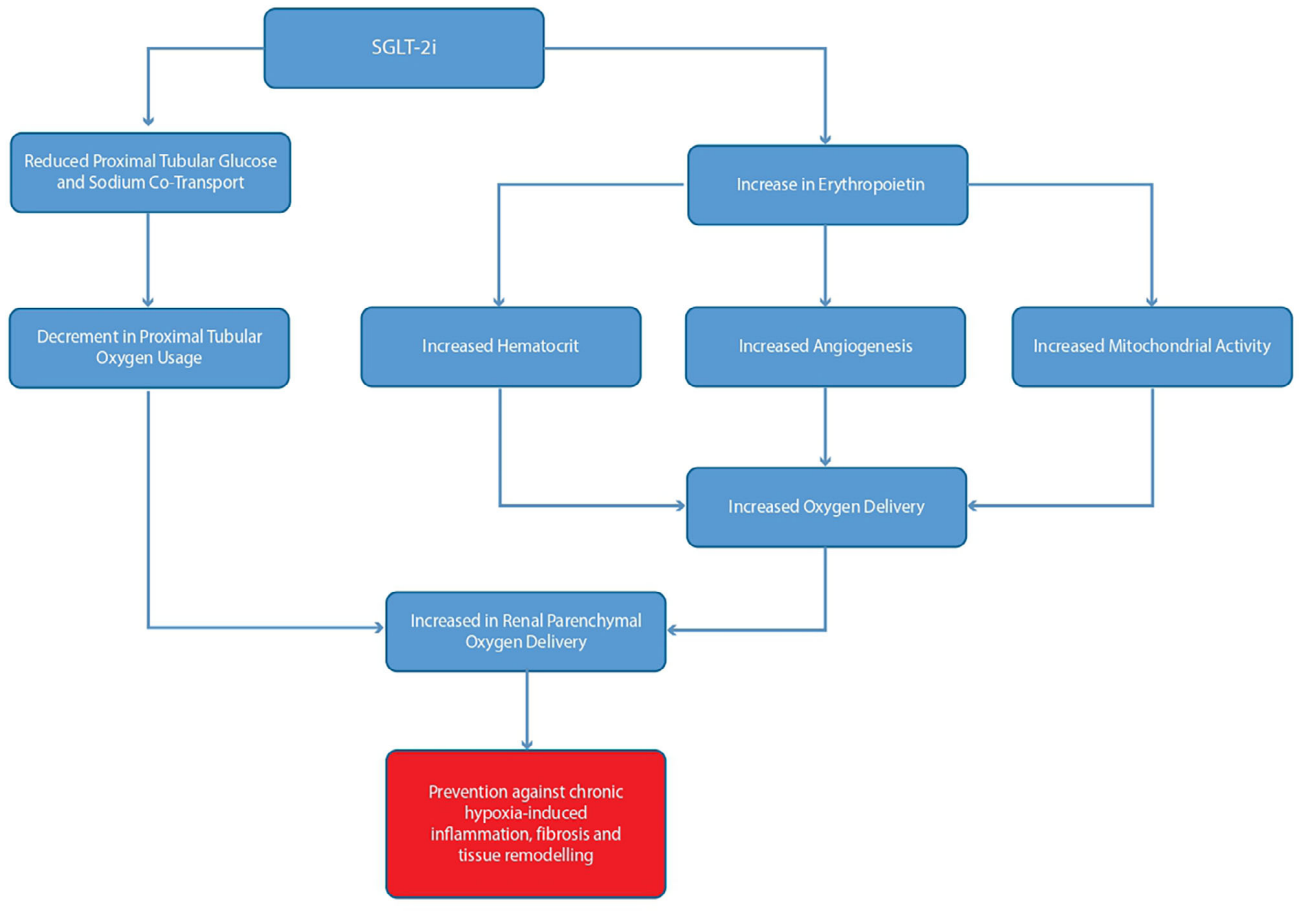
SGLT‐2 inhibition improves renal oxygenation and mitigates chronic hypoxia. SGLT‐2 inhibitors reduce proximal tubular oxygen consumption by limiting glucose and sodium reabsorption, enhancing oxygen availability in renal tissue. Increased erythropoietin production and hematocrit further improve oxygen delivery. Collectively, these mechanisms alleviate intrarenal hypoxia, reducing inflammation, fibrosis, and tubular injury, thereby promoting graft preservation. EPO, erythropoietin; Hct, hematocrit; RBC, red blood cell; SGLT‐2i, sodium–glucose cotransporter‐2 inhibitors.

By reducing proximal tubular sodium reabsorption and gluconeogenesis, SGLT‐2i lowers renal cortical oxygen consumption [19, 52]. This can improve the oxygen tension in kidney tissue (alleviating intrarenal hypoxia). Enhanced oxygen availability may preserve nephron health and reduce the chronic hypoxia‐driven fibrosis that often follows kidney injury. Improved renal oxygenation could be particularly beneficial in transplanted kidneys, which often suffer ischemia‐reperfusion injury and have marginal oxygen supply in the outer medulla. Indeed, better maintenance of peritubular capillary flow (through the hemodynamic effects) and reduced oxygen demand could synergistically protect the graft from chronic hypoxic damage (Figure 4).

## 6 SGLT‐2i in Transplantation: Clinical Experience in Kidney Transplant Recipients

Despite well‐established renoprotective benefits of SGLT‐2 inhibitors in patients with CKD, KTRs have been systematically excluded from pivotal clinical trials. Consequently, current insights regarding the efficacy and safety of SGLT‐2 inhibitors in the transplant population are derived mainly from retrospective cohorts, small‐scale case series, and a limited pilot randomized controlled trial. In this section, we comprehensively review and discuss existing clinical evidence in KTRs, focusing on graft function, metabolic outcomes, safety considerations, and the potential impact on rejection episodes.

### 6.1 Metabolic Control and Cardiorenal Benefits in KTRs

Post‐transplant diabetes mellitus (PTDM) and pre‐existing diabetes in kidney transplant recipients (KTRs) are associated with adverse graft and patient outcomes. SGLT‐2 inhibitors, through their glucose‐lowering effects, offer potential benefits in managing hyperglycemia in this population. Multiple observational studies and clinical trials have reported modest improvements in glycemic control (HbA₁c reductions of approximately 0.4%–0.5%) and significant weight loss (around 2–3 kg) over 6 to 12 months of therapy (13, 14, 52–56) [12, 13, 53, 54, 55, 56]. For instance, the GREAT‐ASTRE study involving 347 KTRs demonstrated a 0.36% reduction in HbA₁c and a 2.2 kg decrease in body weight over a median follow‐up of 12 months [55]. While some studies did not observe significant changes in systolic blood pressure, others, like the study by AlKindi et al., noted reductions in both body weight and blood pressure without significant adverse effects [53]. A small randomized controlled trial by Halden et al. reported that empagliflozin significantly lowered HbA₁c (∼0.5% greater reduction than placebo) and body weight (∼2.5 kg loss vs. placebo) over 3 months, without causing hypoglycemia or loss of graft function [56]. These findings suggest that SGLT‐2 inhibitors may contribute to improved metabolic profiles and cardiovascular risk factors in KTRs.

Proteinuria reductions have also been observed with SGLT‐2 inhibitors use in KTRs [12, 57, 58]. For example, a Spanish cohort of 339 KTRs (mixed diabetic and nondiabetic, mostly on ACEi/ARB) found significantly decreased proteinuria (urine protein–creatinine ratios) 6 months after SGLT‐2i initiation [57]. Similarly, a retrospective single‐center study reported that albuminuria dropped in SGLT‐2i‐treated KTRs without adverse effects on graft function over 1 year [12].

Kidney transplantation significantly improves cardiovascular outcomes in patients with end‐stage kidney disease, yet cardiovascular disease remains the primary cause of death and graft loss in diabetic KTRs. Although specific cardiovascular outcomes in KTRs are less frequently reported, improved blood pressure, weight, and glycemic control likely confer substantial cardiovascular benefit. In a recent study of 750 diabetic KTRs, treatment with SGLT‐2 inhibitor significantly lowered the incidence of major adverse cardiovascular events (MACE) from 11.8% to 3.9% over 55 months (adjusted HR: 0.30; 95% CI: 0.10–0.88; *p* = 0.028), driven mainly by reductions in myocardial infarction. These findings indicate SGLT2i can effectively decrease cardiovascular risk in diabetic KTRs [59].

Clinical evidence from observational studies and pilot trials indicates that KTRs treated with SGLT‐2 inhibitors typically experience stabilized or modestly improved graft function, as reflected by stable or slightly improved eGFR trajectories compared to control groups. This beneficial effect on graft function, coupled with reduced proteinuria, further supports their potential as adjunctive therapies to standard immunosuppressive regimens.

### 6.2 Allograft Function and Rejection

The ultimate question is whether SGLT‐2 inhibitors’ multitude of effects translate into improved graft longevity or reduced rejection in transplant patients. Given the lack of long‐term randomized data, we must rely on observational signals:

Multiple observational cohorts have demonstrated that kidney transplant recipients treated with SGLT‐2 inhibitors maintain stable graft function in the long term [12, 57, 60]. Importantly, recent evidence indicates that SGLT‐2 inhibitor therapy does not increase acute rejection rates; in fact, some studies suggest these agents may even reduce rejection episodes, highlighting their potential immunomodulatory benefits. For instance, Demir et al. conducted a 1‐year retrospective analysis at two centers (∼50 KTRs on SGLT‐2 inhibitors vs. matched controls). They reported that eGFR in SGLT‐2 inhibitors users initially dipped slightly (presumably due to reduced hyperfiltration) then returned to baseline by 12 months, whereas eGFR in nonusers declined gradually over the year (though the difference was not statistically significant) [12]. Importantly, acute rejection rates were lower in the SGLT‐2i group: 11.1% of SGLT‐2 inhibitors‐treated patients had biopsy‐proven acute rejection within the year, compared to 33.3% in the matched group without SGLT‐2i. This corresponded to an odds ratio of ∼0.24 (indicating ∼76% risk reduction in rejection) with SGLT‐2 inhibitors, which was statistically significant despite the small sample. Furthermore, patients on SGLT‐2 inhibitors who did experience rejection tended to have it later and less severely. In a meta‐analysis, SGLT‐2 inhibitors did not significantly increase the risk of allograft rejection compared to the control, with a RR of 0.53 (95% CI 0.11, 2.44; *p* = 0.23; I^2^  = 31%) across 3 studies, involving 186 patients [61].

Regarding rejection, it is important to note that no study so far has suggested an increase in rejection with SGLT‐2 inhibitors use. This alleviates a theoretical concern: if SGLT‐2i were potently immunosuppressive, they might predispose to infections; if they somehow activated immunity, they might trigger rejection. Neither adverse scenario has been observed. On the contrary, the immunomodulatory effects of SGLT‐2 inhibitors (discussed earlier) may actually be beneficial, selectively reducing pathogenic T‐cell responses.

That said, without randomized trial data, we must interpret the lower rejection rates in some series with caution. Patients selected for SGLT‐2 inhibitors tend to be those with stable graft function and well‐controlled risk factors (selection bias), and they may inherently be a lower‐risk group for acute rejection. It is encouraging, however, that across available reports, allograft outcomes with SGLT‐2i have been at least as good as in controls, and possibly better.

### 6.3 Safety in Kidney Transplant Recipients

The main safety considerations with SGLT‐2 inhibitors in any population include urinary tract infections (UTIs), genital mycotic infections, volume depletion/hypotension, and euglycemic diabetic ketoacidosis (euDKA). In KTRs, these issues need special attention due to immunosuppression and the single‐kidney status.

Reassuringly, the available evidence indicates that **S**GLT‐2 inhibitors are well‐tolerated in kidney transplant patients, with safety profiles similar to those seen in nontransplant cohorts:

**UTIs**: Despite initial concerns, current data suggest that UTI rates in KTRs receiving SGLT‐2 inhibitors are comparable to rates in nontransplant diabetic cohorts [12, 13, 61]. Ramakrishnan et al. reviewed the safety profile of SGLT‐2 inhibitors in KTRS, highlighting UTIs as the most common adverse event, with reported incidences ranging widely (0%–36%), though most consistently between 13% and 15% [13]. Genital infections occurred less frequently, and serious complications such as diabetic ketoacidosis, Fournier's gangrene, or amputations were notably absent, suggesting a generally favorable infection‐related safety profile for SGLT2 inhibitors in this population. Demir et al. found a 16% UTI prevalence at 1 year, with only one discontinuation due to recurrent UTIs [12]. In a multicenter retrospective cohort of 339 KTRs initiating SGLT‐2 inhibitors therapy, UTIs were the most commonly reported adverse event, occurring in 14% of patients within 6 months; genital infections were less frequent [57]. In a multicenter study of 347 KTRs on SGLT‐2 inhibitors, the incidence of UTIs was only 6.6% over a year, and no serious urosepsis or pyelonephritis cases were attributed to SGLT‐2 inhibitors [55]. This UTI rate was comparable to or lower than some historical rates in KTRs not on SGLT‐2 inhibitors. A meta‐analysis of 132 diabetic KTRs reported 14 UTIs cases [62].
**Genital mycotic Infections**: In KTRs, the reported rate of genital infections on SGLT‐2 inhibitors has been quite low. For example, Halden et al. (RCT in KTRs) noted one case of genital candidiasis out of 22 treated KTRs [56]. The French study reported a 0.6% incidence (2 out of 347 patients) of genital fungal infection, and the Spanish multicenter study reported ∼1.5% at 1 year – both remarkably low [55, 57]. A meta‐analysis across studies found just 1 case of genital mycosis (∼0.8%) [62]. These numbers are actually lower than what might be expected even in the general SGLT‐2 inhibitor‐treated population. Importantly, no cases of Fournier's gangrene (necrotizing fasciitis of the perineum) have been reported in the transplant literature to date related to SGLT‐2 inhibitors.
**Volume depletion and hemodynamic issues**: SGLT‐2i have a mild diuretic effect (via osmotic diuresis and natriuresis). In transplant patients, especially those on diuretics or with borderline low blood pressure, this could potentially cause orthostatic hypotension or acute kidney injury from volume contraction. The reports indicate that symptomatic hypotension is rare; most KTRs maintain stable blood pressures or even may benefit from a slight reduction in hypertension. No significant increase in acute kidney injury has been linked to SGLT‐2 inhibitors in KTRs except for a drop in eGFR following initiation of SGLT2i, similar to the nontransplant population [14, 56]. Nonetheless, prudent practice is to educate patients to stay well‐hydrated and temporarily hold the SGLT‐2i during any episode of severe gastroenteritis, vomiting, or poor oral intake to avoid prerenal azotemia (the so‐called “sick day” rule).
**Euglycemic DKA**: Although eDKA remains a theoretical concern with SGLT‐2 inhibitors, current evidence does not suggest an increased risk of eDKA among KTRs [12, 13]. A recent review analyzing data from 186 KTRs across three studies found no reported cases of eDKA in this population [61].
**Drug–drug interactions with immunosuppressants**: KTRs commonly receive immunosuppressive medications such as tacrolimus, cyclosporine, sirolimus, everolimus, mycophenolate mofetil, corticosteroids, and belatacept. Current evidence consistently indicates that SGLT‐2 inhibitors do not cause clinically significant pharmacokinetic interactions with these immunosuppressants [12, 56, 63, 64]. Specifically, empagliflozin was found not to affect the trough levels of tacrolimus, cyclosporine, or everolimus, as reported by Halden et al., while Shah et al. similarly showed stable tacrolimus levels with canagliflozin treatment [56, 64]. Mechanistically, this lack of interaction is likely due to minimal involvement of SGLT‐2 inhibitors in cytochrome P450 (CYP) enzyme activity; for example, canagliflozin shows only weak inhibitory effects on CYP2B6, CYP2C8, CYP2C9, and CYP3A4 in vitro [65]. Beyond pharmacokinetic compatibility, SGLT‐2 inhibitors may also confer renal protective effects when used with nephrotoxic immunosuppressants. Jin et al. demonstrated that empagliflozin protected against tacrolimus‐induced renal injury by reducing albuminuria, histological kidney damage, oxidative stress, and apoptotic cell death, further highlighting potential therapeutic synergy [66]. Additionally, corticosteroids and belatacept show no metabolic interactions with SGLT‐2 inhibitors, indicating that these agents can be safely co‐administered without necessitating immunosuppressive dosage adjustments.

### 6.4 Current Status and Future Directions

Current evidence supporting SGLT‐2 inhibitor use in KTRs is predominantly derived from observational studies and small pilot trials, which inherently have limitations such as selection and survivor biases, short follow‐ups, and lack of control groups, precluding robust causal inference or assessment of long‐term outcomes. Prospective randomized controlled trials are critically needed to clarify the impact of SGLT‐2 inhibitors on graft function decline, proteinuria, biopsy‐proven histological injury, acute rejection rates, and cardiovascular events, given KTRs' high baseline cardiovascular risk. Encouragingly, randomized trials are now being planned, which should definitively address these pivotal clinical endpoints. Meanwhile, ongoing observational studies provide supportive preliminary safety and efficacy data, guiding cautious off‐label use and establishing a foundation for future large‐scale prospective research.

## 7 Conclusion

SGLT‐2 inhibitors represent a promising adjunctive therapy in KTRs, potentially enhancing graft survival and overall recipient health. Current evidence from mechanistic research, landmark CKD trials, and observational studies in KTRs highlights their benefits beyond glycemic control, including reduced glomerular hyperfiltration, mitigation of inflammatory and fibrotic processes, and possible modulation of immune responses. Clinically, SGLT‐2 inhibitors have been associated with stable or improved graft function, decreased proteinuria, better blood pressure and weight management, and no increased risk of rejection. Their favorable safety profile, including no significant rise in infections or adverse drug interactions, further supports their use. While definitive long‐term benefits like reduced allograft failure or biopsy‐proven rejection require prospective trials, early findings, such as those from a large French cohort, are encouraging. Current data already justify cautious integration of SGLT‐2 inhibitors into clinical practice for selected KTRs, addressing metabolic and immunologic challenges to improve long‐term transplant outcomes.

## Conflicts of Interest

The authors declare no conflict of interest.
